# Integrated identification of key immune related genes and patterns of immune infiltration in calcified aortic valvular disease: A network based meta-analysis

**DOI:** 10.3389/fgene.2022.971808

**Published:** 2022-09-21

**Authors:** Li-Da Wu, Feng Xiao, Jin-Yu Sun, Feng Li, Yu-Jia Chen, Jia-Yi Chen, Jie Zhang, Ling-Ling Qian, Ru-Xing Wang

**Affiliations:** ^1^ Department of Cardiology, Wuxi People’s Hospital Affiliated to Nanjing Medical University, Wuxi, China; ^2^ Department of Cardiology, The First Affiliated Hospital of Nanjing Medical University, Nanjing, China

**Keywords:** immune-related genes, calcific aortic valve disease, immune cells, immune infiltration, CIBERSORT

## Abstract

**Background:** As the most prevalent valvular heart disease, calcific aortic valve disease (CAVD) has become a primary cause of aortic valve stenosis and insufficiency. We aim to illustrate the roles of immune related genes (IRGs) and immune cells infiltration in the occurrence of CAVD.

**Methods:** Integrative meta-analysis of expression data (INMEX) was adopted to incorporate multiple gene expression datasets of CAVD from Gene Expression Omnibus (GEO) database. By matching the differentially expressed genes (DEGs) to IRGs from “ImmPort” database, differentially expressed immune related genes (DEIRGs) were screened out. We performed enrichment analysis and found that DEIRGs in CAVD were closely related to inflammatory response and immune cells infiltration. We also constructed protein–protein interaction (PPI) network of DEIRGs and identified 5 key DEIRGs in CAVD according to the mixed character calculation results. Moreover, CIBERSORT algorithm was used to explore the profile of infiltrating immune cells in CAVD. Based on Spearman’s rank correlation method, correlation analysis between key DEIRGs and infiltrating immune cells was performed.

**Results:** A total of 220 DEIRGs were identified and the enrichment analysis of DEIRGs showed that they were significantly enriched in inflammatory responses. PPI network was constructed and *PTPN11*, *GRB2*, *SYK*, *PTPN6* and *SHC1* were identified as key DEIRGs. Compared with normal aortic valve tissue samples, the proportion of neutrophils, T cells CD4 memory activated and macrophages M0 was elevated in calcified aortic valves tissue samples, as well as reduced infiltration of macrophages M2 and NK cells activated. Furthermore, key DEIRGs identified in the present study, including *PTPN11*, *GRB2*, *PTPN6*, *SYK*, and *SHC1*, were all significantly correlated with infiltration of various immune cells.

**Conclusion:** This meta-analysis suggested that *PTPN11*, *GRB2*, *PTPN6*, *SYK*, and *SHC1* might be key DEIRGs associated with immune cells infiltration, which play a pivotal role in pathogenesis of CAVD.

## Introduction

Calcific aortic valve disease (CAVD), the most common cardiovascular valve disease, has become a major reason for aortic valve stenosis and insufficiency, especially in the elderly (Tsimikas et al., 2018). It is reported that over 30% of individuals beyond the age of 65 have echocardiography evidence of CAVD (Otto and Prendergast, 2014). With the progression of CAVD, aortic valve stenosis affects almost 3% of people over 65 years of age and in nearly 8% of people over 75 years of age. Considering the prolonged life expectancy, the worldwide CAVD burden is projected to be 4.5 million dollars in 2030 (Vahanian et al., 2012). Currently, surgical therapy remains the only effective therapeutic method against CAVD, which is limited in terms of high costs, perioperative complications, and the complications of life-long anticoagulation therapy (Myasoedova et al., 2018).

CAVD is a progressive disease, including three stages. Valve endothelial cells injury, lipid deposition, and inflammation constitute an initiation stage. In the next stage, valve interstitial cells differentiation and microcalcification are promoted by collagens and bone-matrix proteins deposition. Finally, valvular osteogenesis occurs through activation of various specific molecular signals (Liu and Xu, 2016). Recently, inflammation and immunity has been found to be important to the progression of CAVD. In the aortic valve, nearly 15% of the cells come from hematopoietic sources. With the infiltration of T lymphocytes, B lymphocytes and macrophages into the aortic valve after inflammation, this number increases greatly, so as to promote further inflammation response (Bartoli-Leonard et al., 2021). It is of great value to evaluate immune cells infiltration and find key immune related genes (IRGs) that regulate the infiltration of immune cells for elucidating the molecular mechanism of CAVD.

Integrative meta-analysis of expression data (INMEX) has been widely used in integrating gene expression profiles (Xia et al., 2013). In the present meta-analysis, INMEX was adopted to integrate all datasets of CAVD from Gene Expression Omnibus (GEO) database (GSE12644, GSE83453, and GSE51472) and identify differentially expressed genes (DEGs) in CAVD. Subsequently, we screened out differentially expressed immune related genes (DEIRGs) through matching 2,484 IRGs from ImmPort database to DEGs (Fu et al., 2021). Gene ontology (GO) and kyoto encyclopedia of genes and genomes (KEGG) analysis was conducted to explore the biological meaning of the DEIRGs and the immune-related molecular mechanisms underlying CAVD. CIBERSORT, a widely used algorithm, can assess infiltrating immune cells according to different gene expression patterns (Newman et al., 2015). Accumulating studies have adopted CIBERSORT to evaluate immune cells infiltration in many different diseases (Zhang et al., 2019; Deng et al., 2020; Liu et al., 2020). CIBERSORT was firstly used to investigate the infiltration of 22 immune cells in aortic valve tissue samples from patients with CAVD in this meta-analysis. In addition, we constructed protein-protein interaction (PPI) network and identified key DEIRGs of CAVD. The correlation between each key DEIRGs and infiltrating immune cells was studied respectively to explore its role in CAVD.

## Materials and methods

### Inclusion of eligible datasets

We conducted literature search in GEO database. Search keywords were “CAVD” or “calcific aortic valve disease” or “aortic valve calcification” containing in all fields. A total of 68 researches were screened out. Two independent researchers (Jia-Yi Chen and Li-Da Wu) searched and reviewed the titles, abstracts, and full texts to determine the inclusion. The inclusion criteria are as follows: 1) adult patients with CAVD; 2) at least 6 samples included in each group; 3) genomic data of patients with CAVD and normal individuals were detected by microarray or next generation sequencing. As shown in Table 1, all of the datasets of CAVD in GEO database were included in our meta-analysis, including GSE12644 (Bossé et al., 2009), GSE51472 (Ohukainen et al., 2015), and GSE83453 (Guauque-Olarte et al., 2016). The CAVD microarray datasets in GEO database (Barrett et al., 2013) were downloaded via “*GEO query*” package in R 3.6.3 software (Davis and Meltzer, 2007). Stenotic aortic valve tissue samples without calcification were excluded for the accuracy of the present meta-analysis focusing on CAVD. GSE12644, based on GPL570 platform, includes 10 aortic valve samples from normal individuals and 10 aortic valve samples from patients with CAVD (Bossé et al., 2009). GSE51472, also performed by GPL570 platform, includes 5 aortic valve samples from normal individuals and 5 aortic valve samples from patients with CAVD (Ohukainen et al., 2015). GSE83453, based on GPL10558, includes 8 aortic valve samples from normal individuals and 10 aortic valve samples from patients with CAVD (Guauque-Olarte et al., 2016). The basic information of the patients included in this meta-analysis was also downloaded from the GEO database. Considering the difficulty of obtaining aortic valve samples in the clinic and the age-dependent and gender-dependent clinical features of CAVD, all of the aortic valve samples included in this meta-analysis were derived from elderly male individuals. The mean age of patients in the control group was 58.8 ± 2.01 years, and that in the CAVD group was 62.8 ± 1.48 years, the difference was not statistically significant.

**TABLE 1:** Characteristics of the datasets included in the integrated analysis.

GEO ID	Platform	Citation	Region	Normal	CAVD
GSE12644	GPL570; Affymetrix Human Genome U133 Plus 2.0 Array	Bossé Y, et al. Circ Cardiovasc Genet, 2009;2(5):489-498. PMID: 20031625	Quebec, Canada	10	10
		Derbali H, et al. Am J Pathol, 2010;176(6):2,638-2,645. PMID: 20382708			
GSE51472	GPL570; Affymetrix Human Genome U133 Plus 2.0 Array	Ohukainen P, et al. Ann Med, 2015;47(5):423-429. PMID: 26203686	Oulu, Finland	5	5
		Rysä J, et al. Genom Data, 2016;7:107-108. PMID: 26981379			
GSE83453	GPL10558; Illumina HumanHT-12 V4.0 expression beadchip	Guauque-Olarte S, et al. Physiol Genomics, 2016;48(10):749-761. PMID: 27495158	Quebec, Canada	8	10

GEO, gene expression omnibus; CAVD, calcific aortic valve disease.

### Quality assessment and removal of batch effects among different datasets

Log2-transformation and background correction were performed on the gene expression profiles using the “linear models for microarray data (*limma*)” package (Ritchie et al., 2015). After the normalization process, all of the microarray probes were translated to official gene names in INMEX. For multiple probes that detected a single gene, we use their average expression values. In the era of omics and big data, the integration of data (the same disease or condition) tested in different batches, platforms, using different techniques, and under different laboratory conditions will become the norm. However, different batches of datasets may have batch effects due to abiotic factors, which may have a serious impact on the test results and even lead to wrong conclusions. There are several methods for removing the batch effect of gene expression data, including ComBat method, surrogate variable analysis method, distance weighted discriminant method and ratio-based method. Considering the datasets included in the present meta-analysis were based on different platforms and different experimental conditions, ComBat option was used to remove batch effect and visualize the results of principal component analysis (PCA). Moreover, each gene expression value from different batches were adjusted by the normalization procedure of “central standardization,” also known as “mean centering.” The specific method of “central standardization” is to subtract from the mean value of each gene so that the mean value of each gene expression value in the transformed dataset was 0. Through the normalization procedure of “central standardization,” gene expression values were transformed to the appropriate range so as to avoid the fluctuation of small value variables being masked by large value variables.

### Network based meta-analysis and identification of differentially expressed immune related genes

Following the PRISMA guidelines (Moher et al., 2009), INMEX was used to integrate gene expression datasets of CAVD through network-based meta-analysis (Xia et al., 2013). Moreover, the random effect model was selected in this study considering the heterogeneity among different datasets (Xia et al., 2013). Pattern extractor tool in INMEX was used to construct a heatmap of the top 100 DEGs. By matching 2,484 IRGs from the ImmPort database to DEGs, we screened out DEIRGs in aortic valve tissue samples from patients with CAVD (Li et al., 2014).

### Assessment of immune cells infiltration

In this meta-analysis, CIBERSORT algorithm was firstly used to assess the infiltration of 22 types of immune cells in aortic valve tissue samples from patients with CAVD (Newman et al., 2015). Actually, CIBERSORT algorithm has been employed to evaluate immune cells infiltration in many different diseases, such as osteoarthritis (Deng et al., 2020), high-grade serous ovarian cancer (Liu et al., 2020), and breast ductal and lobular carcinoma (Zhang et al., 2019). For the accuracy of evaluation, the *p* value of CIBERSORT results adopted in the present study are less than 0.05. In each sample, the proportions of various immune cells were visualized using R software. Moreover, we also carried out PCA analysis based on the dataset of relative fractions of immune cells infiltration in each sample calculated by CIBERSORT (rows: the relative fractions of immune cells infiltration; columns: aortic valve tissue samples). PCA analysis adopted the method of multivariate statistical distribution analysis with characteristic quantities. Generally, this operation can be regarded as a method to expose the internal structure of data so as to better explain the variables of data. Of note, principal component analysis used orthogonal transformation to linearly transform the specific data values of immune cell infiltration, and then projected them into the values of multiple linearly uncorrelated variables. Samples were clustered according to the values of the first two linearly uncorrelated variables. “*ggplot2*” package in R software was adopted to perform PCA analysis based on immune cells infiltration and draw a PCA clustering plot. A correlation heatmap was created by “*corrplot*” package, also based on the dataset of relative fractions of immune cells infiltration in each sample calculate by CIBERSORT, to describe the correlation between 22 types of immune cells, correlation coefficient and *p* value were used to evaluate the strength and significance of correlation. For a specific type of immune cell, the difference in immune cells infiltration levels between aortic valve samples from patients with CAVD and normal individuals were represented by a violin plot established by “*vioplot*” package.

### Enrichment analysis of differentially expressed immune related genes

To explore the biological functions of DEIRGs and roles of DEIRGs in immune cells infiltration in CAVD, the “*clusterProfler*” package (Wu et al., 2021) was adopted to conduct GO and KEGG pathway enrichment analysis. The enrichment terms were rendered as a network plot and visualized by Metascape software for elucidating the correlation among them (Zhou et al., 2019). Enrichment analysis was also performed based on DisGeNET (Pinero et al., 2017) and TRRUST (Han et al., 2018) database to further explore roles of DIREGs in CAVD. DisGeNET is a database of gene-disease associations, which collects one of the largest publicly available collections of genes and human diseases-related variants. TRRUST is a visual and manually annotated transcriptional regulatory network database. TRRUST not only contains target genes corresponding to transcription factors, but also contains regulatory relationships among transcription factors.

### Protein–protein interaction network and identification of key differentially expressed immune related genes

The STRING database is a widely used database for protein-protein interactions (PPIs). This database can be applied to 2031 species, containing 9.6 million proteins and 13.8 million PPIs. At present, STRING database is widely used to study the interaction network between proteins, which helps to find the core regulatory genes in PPIs network (Zhao et al., 2018; Bajpai et al., 2020; Liu et al., 2021). The STRING software (Szklarczyk et al., 2019) was adopted to construct PPI network of DEIRGs, which was visualized by Cytoscape software 3.8.1 (Shannon et al., 2003). According to previously published studies, we chose the confidence value of 0.9 and the maximum number of connections of 3 to screen out relatively reliable protein interaction relationships on the basis of preserving protein correlations as much as possible (Wang et al., 2021; Ramadhani et al., 2022; Yadalam et al., 2022). Cytohubba is a plug-in of Cytoscape software for identifying hub gene nodes (Chin et al., 2014). It provides multiple analysis algorithms to calculate hub genes in protein interaction network diagrams. Among them, the mixed character calculation algorithm is a relatively accurate method that has been proved to predict important targets (Chin et al., 2014). Mixed character calculation algorithm is a method to judge the importance of hub genes by evaluating the node degree, betweenness centrality in the PPI network. The specific calculation method of MCC is as follows: given a node v, the MCC of *v* is defined as $MCC\;\left(v\right)=\sum_{C\in S\left(v\right)}\left(\left|C\right|-1\right)!$ , where S(v) is the collection of maxima l cliques which contain *v*, and (|C|-1)! is the product of all positive integers less than |C|. If there is no edge between the neighbors of the node *v*, then MCC(*v*) is equal to its degree (Chin et al., 2014). Furthermore, using Cytoscape plugin software “*cytoHubba*,” the top 5 hub DEIRGs were screened out based on mixed character numeration.

### Correlation analysis between key differentially expressed immune related genes and infiltrating immune cells

**FIGURE 1:**
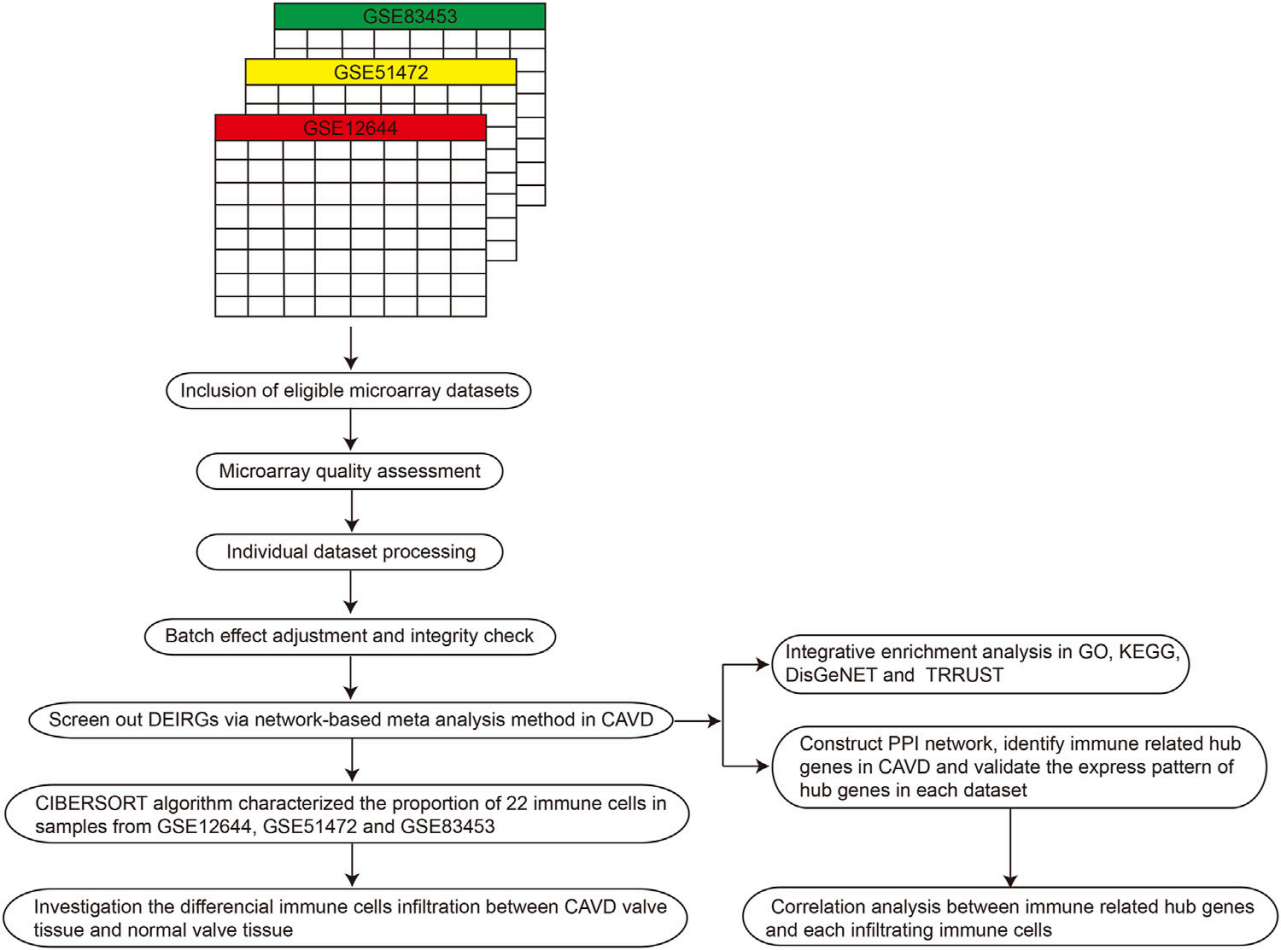
Workflow of the present systematic review and meta-analysis. PPI, protein–protein interaction; GO, gene ontology; CAVD, calcific aortic valve disease; DEIRGs, differentially expressed immune related genes; KEGG, kyoto encyclopedia of genes and genomes.

The correlation between the expression values of key DEIRGs and the relative fractions of immune cells infiltration was analyzed using spearman method in R software, and the package of “*ggplot2*” was employed to visualize the correlation analysis results Figure 1.

## Results

### Differentially expressed genes and differentially expressed immune related genes screening between calcified and normal aortic valves

**FIGURE 2:**
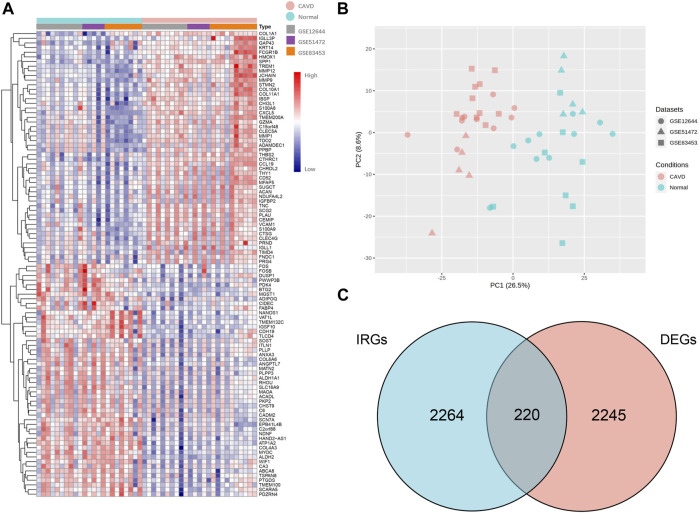
Identification of DEIRGs between aortic valve samples from patients with CAVD and normal individuals through network-based meta-analysis. **(A)** Heatmap of top 50 up-regulated DEIRGs and top 50 down regulated DEIRGs across different datasets (according to fold changes), hierarchal clustering is applied based on complete linkage method. **(B)** PCA plot after removing batch effect between GSE12644, GSE51472 and GSE83453. **(C)** Venn plot of screening DEIRGs by matching the 2,484 IRGs from ImmPort database to the 2,465 DEGs. CAVD, calcific aortic valve disease; IRGs, immune related genes; DEGs, differentially expressed genes; DEIRGs, differentially expressed immune related genes.

We performed principal component analysis (PCA) to evaluate whether the batch effects were successfully removed among different datasets included in the present meta-analysis. In Figure 2B, the PCA plot demonstrated that batch effect among GSE12644, GSE83453 and GSE20681 was successfully removed. In INMEX, random effect model was used to identify DEGs according to the adjusted *p* value < 0.05. A total of 2,465 DEGs were screened out, including 1306 up-regulated genes and 1159 down-regulated genes in aortic valve tissues from patients with CAVD. The top 50 up-regulated DEIRGs and top 50 down regulated DEIRGs across different datasets are shown in a heatmap, hierarchal clustering is applied based on complete linkage method (Figure 2A). A total of 220 DEIRGs were finally selected after matching the DEGs to the IRGs from ImmPort database (Figure 2C).

### Immune cells infiltration analysis

**FIGURE 3:**
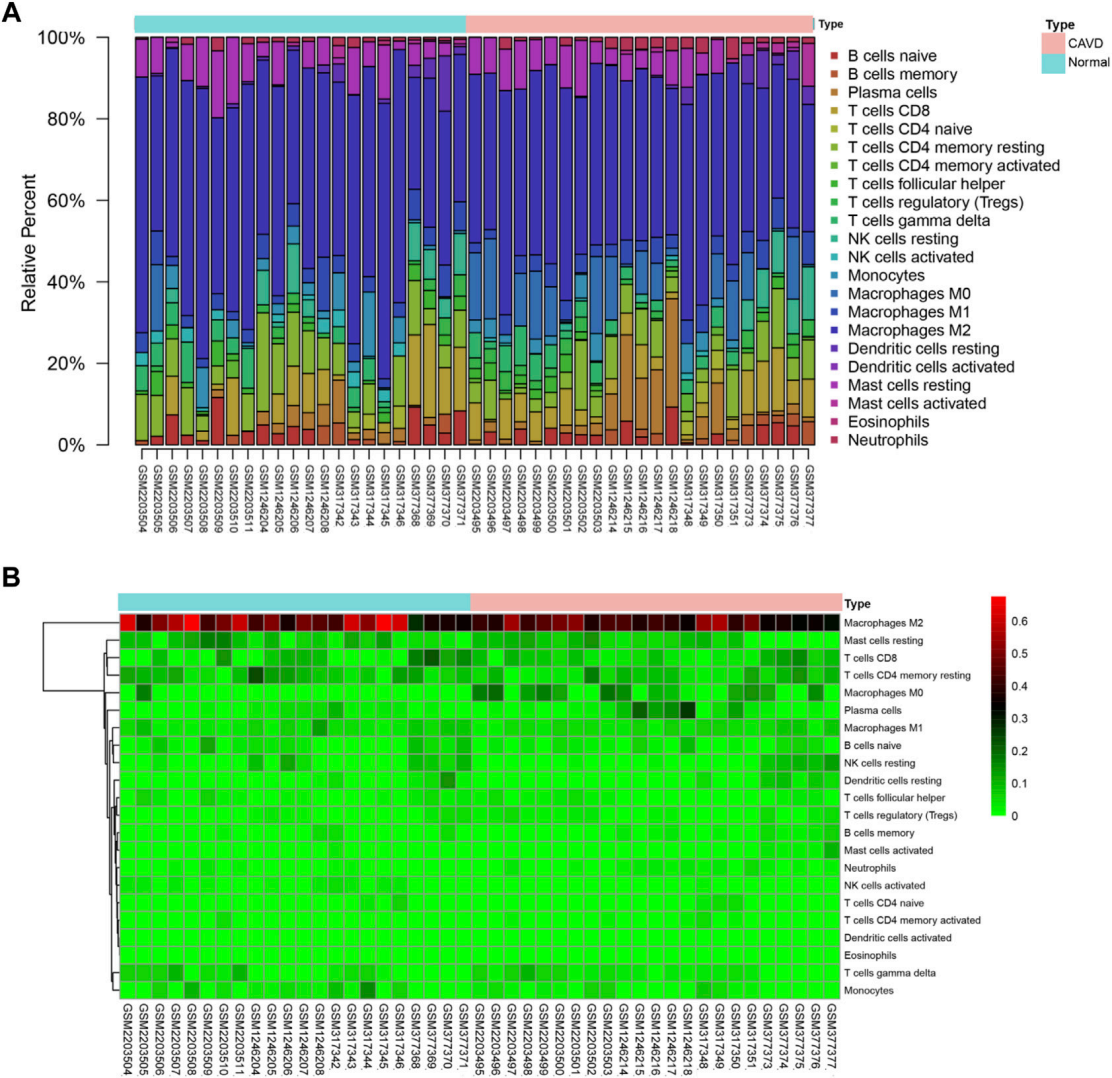
Summary of immune cells infiltration in calcified and normal aortic valve samples. **(A)** Barplot shows the relative fractions of 22 types of immune cells in each sample. **(B)** Heatmap of the relative fractions of 22 subpopulations of infiltrating immune cells in each sample, green to red indicates an increase in relative fractions of immune cells infiltration. CAVD, calcific aortic valve disease.

**FIGURE 4:**
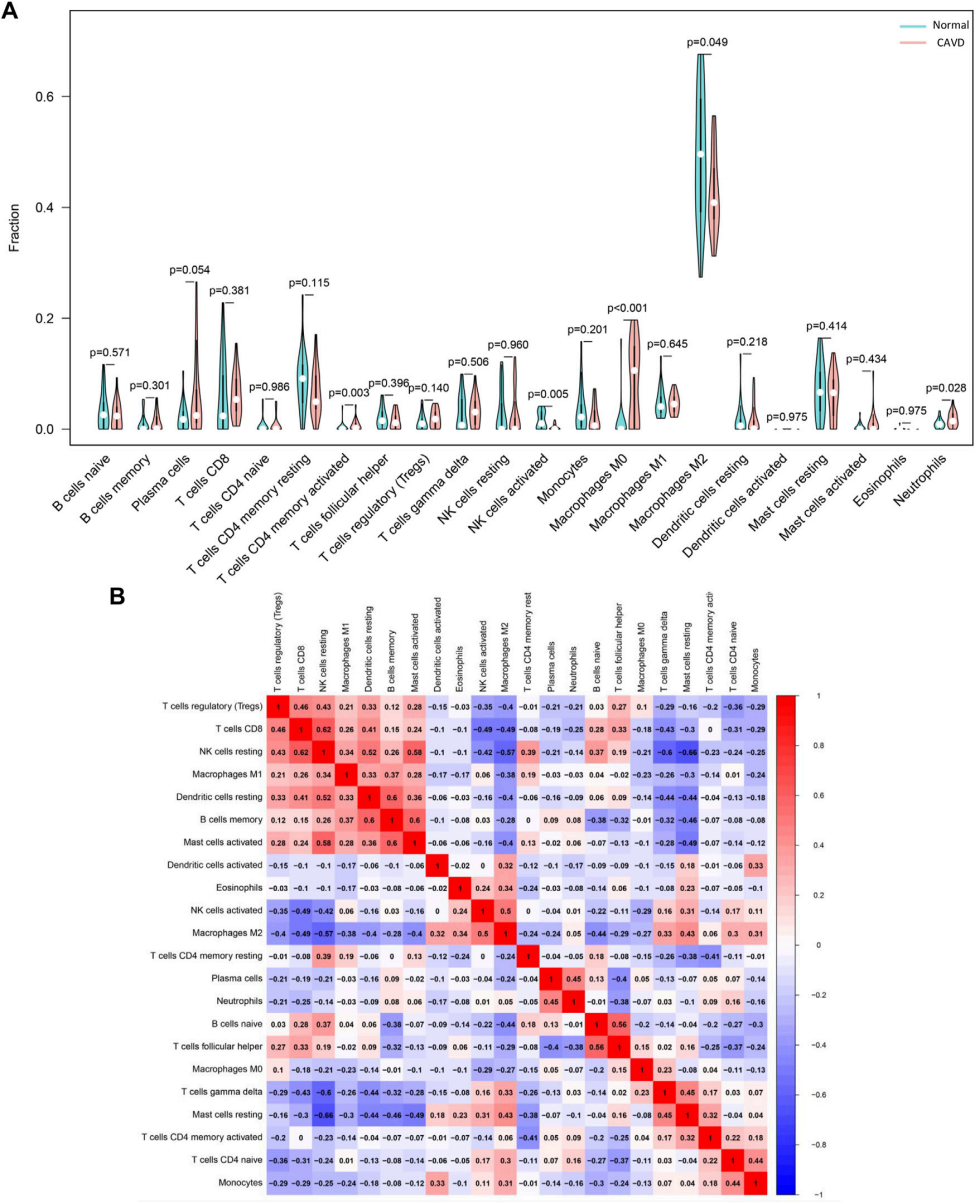
Evaluation of the difference in immune cells infiltration between aortic valve samples from patients with CAVD and normal individuals. **(A)** The difference in the relative fractions of 22 subpopulations of immune cells between calcified and normal aortic valve samples. **(B)** Correlation heatmap based on the Spearman’s rank correlation method shows the correlation between the relative fractions of 22 immune cells subpopulations, blue to red indicates an increase in correlation coefficient. CAVD, calcific aortic valve disease; PCA: principal component analysis.

Based on CIBERSORT algorithm, we firstly investigated the infiltration of 22 types of immune cells in aortic valve tissues from normal individuals and patients with CAVD. Figure 3A and Figure 3B vividly illustrate the proportion of infiltrating immune cells in aortic valve tissue samples from 23 normal individuals and 25 patients with CAVD. Compared with normal aortic valve tissue samples, the proportion of neutrophils, T cells CD4 memory activated and macrophages M0 was significantly elevated in the calcified aortic valves tissues, as well as reduced infiltration of macrophages M2 and NK cells activated in the calcified aortic valves tissues (Figure 4A). Because of the high proportion of macrophages M2, we have created a separate heatmap and a separate violin plot in supplementary materials excluding macrophages M2 to better visualize the differences in CAVD versus normal samples observed for the other immune cells (Supplementary Figure S3). The results of correlation analysis of different infiltrating immune cells showed that NK cells resting and T cells CD8 have the strongest positive correlation (r = 0.62; Figure 4B). However, mast cells resting and NK cells resting have the most intensive negative correlation (r = -0.66). According to the proportion of infiltrating immune cells, PCA diagram revealed distinct group bias clustering, indicating that immune cells infiltration of patients with CAVD and normal individuals are significantly different (Supplementary Figure S1).

### Enrichment analysis of differentially expressed immune related genes

**FIGURE 5:**
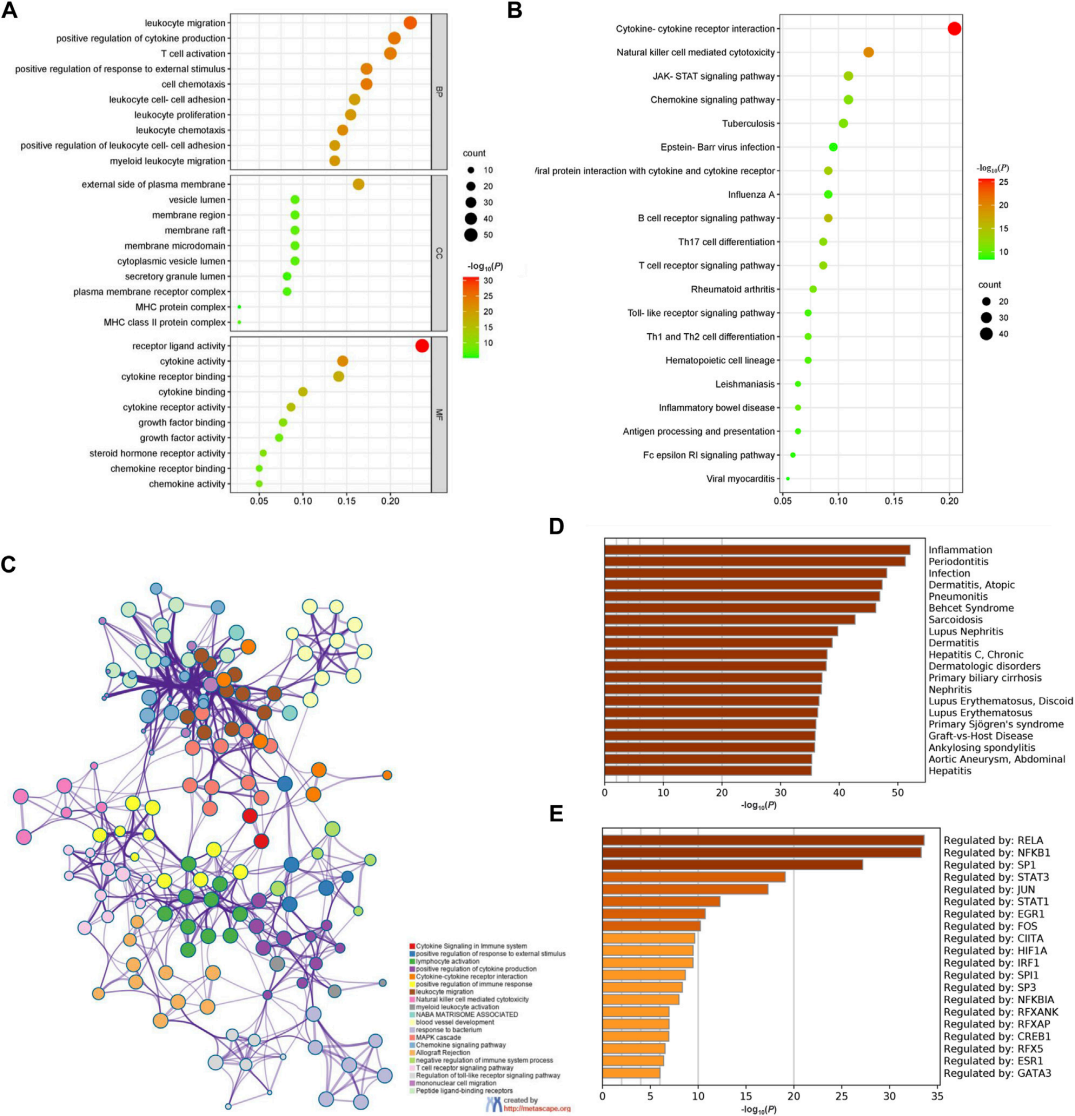
Enrichment analysis of DEIRGs in CAVD. **(A)** GO enrichment analysis. **(B)** KEGG pathway enrichment analysis. **(C)** The network of enriched terms and each node represents an enriched term. **(D)** Summary of enrichment analysis based on DisGeNET database. **(E)** Summary of enrichment analysis based on TRRUST database. The light brown to dark brown gradient indicates an increase in -log_10_(*P*). Count: the number of genes enriched in each term; DEIRGs, differentially expressed immune related genes; GO, gene ontology; KEGG, kyoto encyclopedia of genes and genomes.

We performed enrichment analysis of DEIRGs of CAVD based on GO and KEGG databases. Figure 5A shows that the biological processes were mainly enriched in positive regulation of leukocyte migration, T cell activation, response to external stimulus, cytokine production and cell chemotaxis. And the most enriched cellular components included vesicle lumen, membrane region, the external side of plasma membrane, membrane raft, and membrane microdomain. The molecular functions were mainly enriched in cytokine activity, cytokine binding, cytokine receptor binding, receptor-ligand activity and cytokine receptor activity. In Figure 5B, KEGG analysis shows that NK cell mediated cytotoxicity and cytokine to cytokine receptor interaction were most enriched, followed by JAK-STAT pathway, chemokine, tuberculosis. The top 20 pathways in KEGG enrichment analysis were shown in Figure 5C, including leukocyte migration, cytokine signaling in the immune system, lymphocyte activation, myeloid lymphocyte activation and T cell receptor signaling pathway. In addition, DisGeNET enrichment analysis also revealed that the DEIRGs were significantly associated with inflammation, periodontitis, infection, dermatitis and pneumonitis (Figure 5D). Then, we screened out transcription factors associated with DEIRGs based on the TRRUST database, including RELA, NFKB1, SP1, STAT3, and JUN (Figure 5E).

### Protein–protein interaction network analysis

**FIGURE 6:**
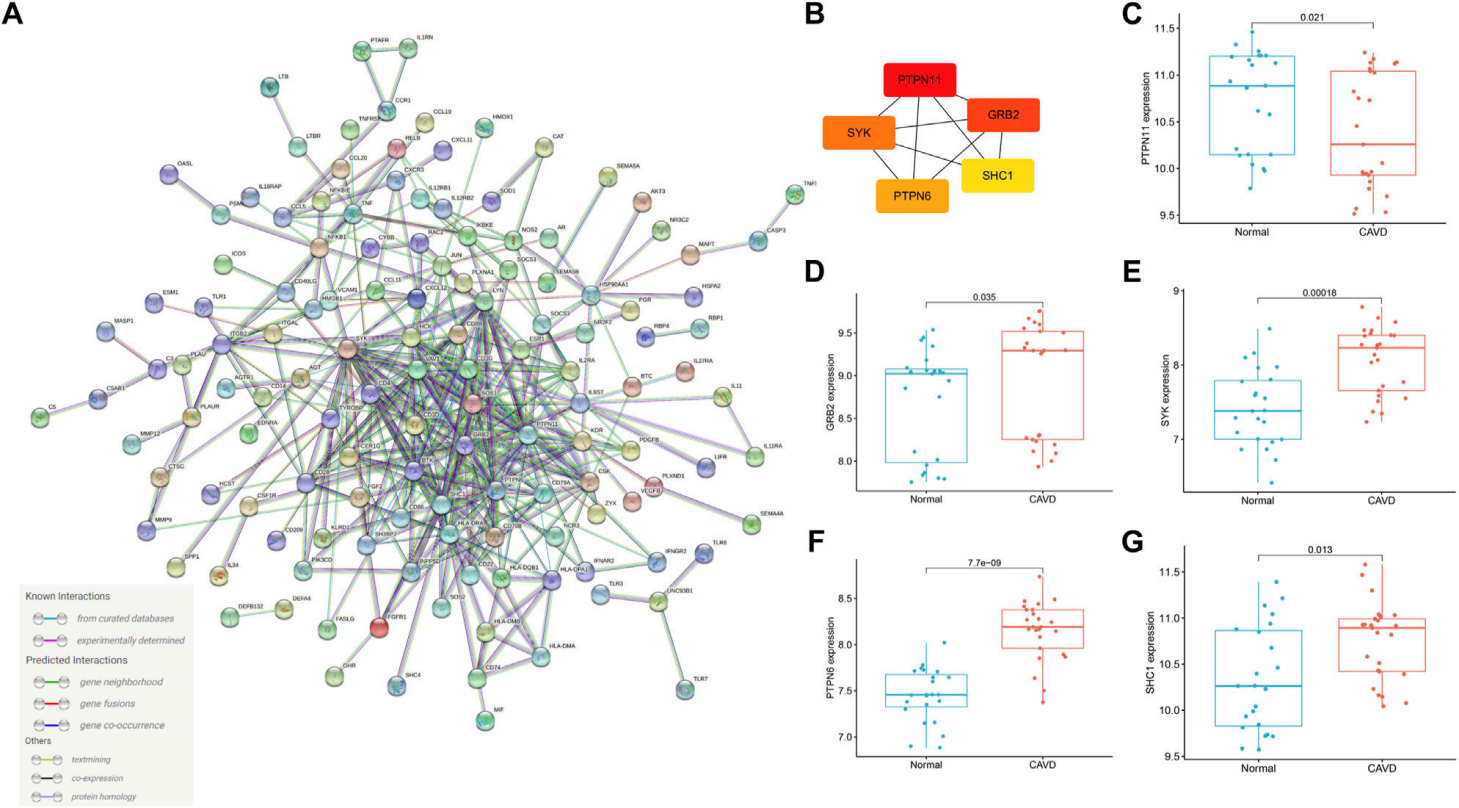
PPI network and identification of hub genes. **(A)** PPI network of DEIRGs in CAVD created by STRING website. The number of edges between different proteins represents the number of evidences supporting the interaction relationship in STRING database. **(B)** Top 5 hub genes identified by “cytoHubba” according to mixed character calculation and its core network. The essentiality of hub genes increases from yellow to red. **(C–G)** The expression of *PTPN11, GRB2, SYK, PTPN6* and *SHC1* in calcified and normal aortic valve. PPI: protein-protein interaction; DEIRGs: differentially expressed immune related genes; CAVD, calcific aortic valve disease.

Figure 6A is the PPI network of DEIRGs, all of the 220 DEIRGs were included in the PPI network, and there are 384 direct interactions. We also carried out an analysis of our PPI network in the STRING database, the results were as followed: number of nodes in the PPI network: 220; number of edges in the PPI network (not the direct interactions but the number of evidence supporting the interactions): 1931; expected number of edges out of a set of randomly selected degree-matched genes: 500; PPI enrichment *p* value: < 0.001. Thus, the interactions among the 220 DEIRGs in this study were more significant than the interactions among a randomly selected set of genes that were matched in degree. In addition, the high interconnectivity between nodes in the PPI network indicates functional cohesion among proteins. Therefore, there are a large number of interactions among the 220 DEIRGs, which may play important roles in various biological processes leading to the development of CAVD. CytoHubba software was adopted to identify the top 5 key DEIRGs according to the core PPI network, including *PTPN11*, *GRB2*, *SYK*, *PTPN6*, and *SHC1* (Figure 6B). As can be seen in Figure 6C, *PTPN11* was statistically down-regulated in the aortic valve tissues from patients with CAVD. Whereas *GRB2*, *SYK*, *PTPN6* and *SHC1* were statistically up-regulated in aortic valve tissues from patients with CAVD (Figures 6D–G).

### Correlation analysis of key differentially expressed immune related genes and immune cells infiltration

**FIGURE 7:**
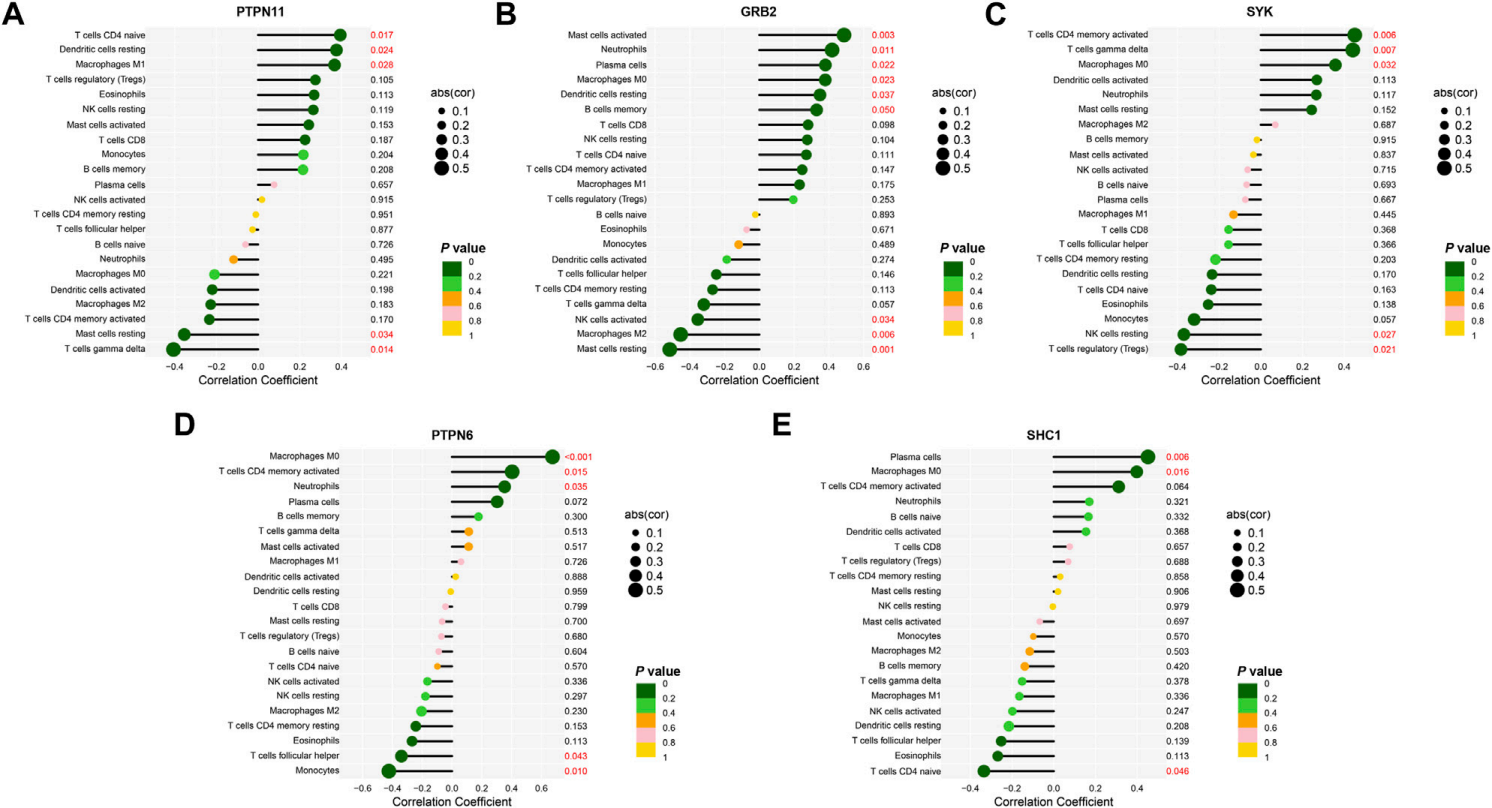
Correlations between the expression values of key DEIRGs and the relative fractions of immune cells infiltration. **(A–E)** Correlation analysis of the association between the expression values of *PTPN11*, *GRB2*, *SYK*, *PTPN6*, *SHC1* and the relative fractions of immune cells infiltration based on Spearman’s rank correlation method.

Results of the correlation analysis between the key DEIRGs and infiltrating immune cells in aortic valve tissues indicated that *PTPN11* was intensively correlated with T cells CD4 naive (r = 0.394, *p* = 0.017), dendritic cells resting (r = 0.376, *p* = 0.024), macrophages M1 (r = 0.367, *p* = 0.028) and negatively correlated with T cells gamma delta (r = −0.406, *p* = 0.014), mast cells resting (r = -0.354, *p* = 0.034). *GRB2* had positive correlation with mast cells activated (r = 0.487, *p* = 0.003), neutrophils (r = 0.418, *p* = 0.011), plasma cells (r = 0.380, *p* = 0.022), macrophages M0 (r = 0.379, *p* = 0.023), B cells memory (r = 0.329, *p* = 0.049), dendritic cells resting (r = 0.349, *p* = 0.037) and negative correlation with NK cells activated (r = −0.354, *p* = 0.034), macrophages M2 (r = -0.453, *p* = 0.005), mast cells resting (r = -0.515, *p* = 0.001). Of note, out of all the gene-immune cell infiltration correlations, the one between GRB2 and resting mast cells seems to be more important. *SYK* had positive correlation with T cells CD4 memory activated (r = 0.450, *p* = 0.006), T cells gamma delta (r = 0.441, *p* = 0.007), macrophages M0 (r = 0.358, *p* = 0.032) and negative correlation with NK cells resting (r = -0.369, *p* = 0.026), Tregs (r = -0.383, *p* = 0.021). *PTPN6* was positively correlated with T cells CD4 memory activated (r = 0.403, *p* = 0.014), neutrophils (r = 0.353, *p* = 0.034), macrophages M0 (r = 0.674, *p* < 0.001) and correlated negatively with T cells follicular helper (r = −0.339, *p* = 0.042), monocytes (r = −0.423, *p* = 0.009). *SHC1* had positive correlation with plasma cells (r = 0.452, *p* = 0.006), macrophages M0 (r = 10.398, *p* = 0.016) and negative correlation with T cells CD4 naive (r = −0.335, *p* = 0.046) (Figure 7).

## Discussion

CAVD, a chronic progressive disease, develops gradually from valvular sclerosis to valvular calcification, eventually leads to stenosis of left ventricular outflow and severely disrupts hemodynamics (Büttner et al., 2021). CAVD has become a major health problem due to its high prevalence, high morbidity and mortality rate. Due to the lack of effective drugs, aortic valve replacement (AVR) or transcatheter aortic valve implantation (TAVI) are the only available treatments for patients with CAVD (Myasoedova et al., 2018). AVR is the traditional treatment for aortic valve disease. However, AVR alone has a high in-hospital mortality rate, approximately 3.4%. Despite TAVI becoming increasingly useful, even for patients at low risk, most patients who undergo it are elderly and frail and have a number of comorbid conditions. The perioperative management of TAVI still presents great challenges. More and more proofs verified that the pathological process involved in CAVD is multifactorial, including aortic valve endothelial cells damage, aortic valve fibrosis and aortic valve calcification. Studies have demonstrated that inflammatory response plays a pivotal role in development of CAVD (Cho et al., 2018; Sikura et al., 2020).

One promising and rapidly evolving tactic to CAVD is the application of multi-omics approaches to fully define disease pathogenesis (Blaser et al., 2021). More and more researchers have focused on changes in gene expression profiles in patients with CAVD. Qiao et al. and Teng et al. investigated the potential DEGs and pathways related to CAVD based on traditional bioinformatic analysis (Teng et al., 2020; Qiao et al., 2022). In addition, based on the WGCNA method, Sun et al. screened out different functional gene modules related to CAVD. However, roles of DEGs in the occurrence and development of CAVD has not been further discussed, especially the relationship between DEIRGs and immune cells infiltration (Sun et al., 2021). In the present study, we aim to screen out key DEIRGs of CAVD based on network bioinformatic analysis and explore the profile of infiltrating immune cells in aortic valve tissues from patients with CAVD in detail.

A total of 220 DEIRGs were identified in aortic valve tissue samples from patients with CAVD after a detailed analysis of all relevant datasets of CAVD in GEO database. GO analysis of the DEIRGs revealed that leukocyte migration, receptor-ligand activity, leukocyte cell-cell adhesion, myeloid leukocyte migration and membrane raft and membrane microdomain were significantly enriched. These biological processes and molecular functions were closely related to immune cells infiltration (Cho et al., 2018). Infiltrating immune cells could release inflammatory and fibrotic cytokines and further aggravate inflammatory response. DEIRGs are also involved in the regulation of cytokine receptor activity, cytokine activity and cytokine production in KEGG analysis. The inflammatory factors secreted by invading inflammatory cells, such as IL-1β and NF-κB, can promote extracellular matrix remodeling, lipid deposition, fibrosis, ossification and calcification (Liu and Pravia, 2010). These findings indicate that DEIRGs in CAVD are involved in the inflammatory processes. In the “cytoHubba” plugin, *PTPN11*, *GRB2*, *SYK*, *PTPN6* and *SHC1* were identified as top 5 key DEIRGs according to the results of mixed character calculation.

Protein tyrosine phosphatase (PTP) is a kind of protein phosphatases, including PTPN1, PTPN2, PTPN6, PTP11 and PTPN22. PTPs function in various important biological processes, including cell cycle and cell differentiation, by carrying out phosphorylation and dephosphorylation of tyrosine residues (Pulido et al., 2013). The role of PTPs in inflammatory response and immune cells infiltration was gradually revealed (Zhao et al., 2016; Xiao et al., 2019). In the present study, *PTPN11* was significantly down-regulated, whereas *PTPN6* was up-regulated in aortic valves from patients with CAVD. PTPN11 have already been linked to inflammation response, which can reduce the level of Th1 cytokine through inhibiting the combination of STAT1 and IFN-γ receptor (Tseng et al., 2012). Moreover, studies have already demonstrated that *PTPN11* gene variants are closely associated ulcerative colitis (UC) but not Crohn’s disease (CD) (Spalinger et al., 2015). Moreover, PTPN11 is an important component in growth factor signaling pathway, closely related to Egfr signaling and formation of valve endothelial cells (Chen et al., 2000). Interestingly, patients with *PTPN11* mutation present significantly higher prevalence of pulmonary valve stenosis, named Noonan syndrome (Brasil et al., 2010). In addition, *PTPN11* mutation has also been demonstrated to be harmful to myocardial hypertrophy and cardiac fibrotic remodeling through crosstalking with NF-κB pathway and mTOR signaling (Schramm et al., 2012; Zhou et al., 2020a). PTPN6, another phosphatase of PTPs, specially expressed in the cytoplasm and prevented excessive autoimmunity in IL-1 dependent inflammatory diseases and pyroptosis dependent inflammatory diseases (Speir et al., 2020). Studies have also demonstrated that PTPN6 significantly ameliorates inflammatory disease by decreasing TNF-α, TGF-β and IL-6 (Lin et al., 2020). In addition, PTPN6 is important in preventing the harmful effects of pathogens on the host, which is crucial for successful defense mechanisms against invading microorganisms (Kanwal et al., 2013). PTPN6 is known as an important negative regulator of inflammatory response and significantly down regulated in aortic valve tissues from patients with CAVD Table 2.

**TABLE 2:** Summary of the functions and known contributions of key DEIRGs in CAVD and other inflammatory diseases.

Gene symbol	Full name	Functions and known contributions of key DEIRGs in CAVD and inflammatory diseases
*PTPN11*	Protein tyrosine phosphatase 11	PTPN11 is an important component in growth factor pathway and closely related to formation of valve endothelial cells. Moreover, PTPN11 can reduce the level of Th1 cytokine through preventing combination of STAT1 and IFN-γreceptor. PTPN11 is associated with inflammatory diseases, including pulmonary valve stenosis, ulcerative colitis, inflammation induced-myocardial hypertrophy and cardiac fibrotic remodeling
*GRB2*	Growth factor receptor-bound protein 2	GRB2 mainly functions in activating Egfr tyrosine kinase and its downstream renin-angiotensin system. GRB2 was also involved in the process of development of T cells and Th cells. Studies have demonstrated that GRB2 was significantly up-regulated in aortic valve tissues form CAVD patients
*SYK*	Spleen-associated tyrosine kinase	SYK is a member of the none receptor type tyrosine kinase family and involved in numerous biological functions. As a proinflammatory molecule, SYK has become a crucial biomarker of coronary heart disease. However, the relationship between SYK and aortic valve diseases still remains exclusive
*PTPN6*	Protein tyrosine phosphatase 6	PTPN6 specially expressed in the cytoplasm, it can prevent excessive autoimmunity in IL-1 dependent inflammatory diseases. PTPN6 can ameliorate inflammatory diseases by decreasing TNF-α, TGF-β and IL-6 and prevent the harmful effects of pathogens on the host. PTPN6 is known as an important negative regulator of inflammatory response and down regulated in patients with CAVD.
*SHC1*	Src-homology 2 domain containing 1	SHC1 is a member of SHC family of adaptor proteins. SHC1 functions in production of reactive oxygen species. Oxidative stress can cause inflammation and play an important role in the development of CAVD. SHC1 mediated-reactive oxygen species production is closely related to development of atherosclerosis and coronary heart disease

CAVD, Calcific Aortic Valve Disease.

GRB2 is a 25kD adaptor protein that functions in modulating and integrating signals from cell membrane surface receptors to intracellular effector proteins (Dharmawardana et al., 2006). Studies have demonstrated that *GRB2* was up-regulated in aortic valve tissues from patients with CAVD (Abazyan et al., 2010). GRB2 is best known in the cardiovascular field for activating Egfr tyrosine kinase and its downstream renin-angiotensin system (Tari and Lopez-Berestein, 2001). Recent studies also revealed that GRB2 was involved in the process of development of T cells and Th cells. *GRB2*-knockout animals have reduced T cells and more prone to inflammatory diseases (Radtke et al., 2016).

Spleen-associated tyrosine kinase (SYK), a member of the none receptor type tyrosine kinase family (Alhazmi, 2018). SYK was also involved in numerous biological functions, including cellular adhesion, vascular development, platelet activation and relaying adaptive immune receptor signaling related to immune cells infiltration (Kurosaki, 2000; Correll et al., 2006; Mocsai et al., 2010). As a proinflammatory molecule, SYK has received increasing attention in some diseases. Liang *et al.* demonstrated that SYK was a crucial biomarker and closely related to the occurrence of coronary heart disease (CHD) as an proinflammatory factor (Liang et al., 2019). Researches on the specific role of SYK in CAVD is helpful to better understand the role of inflammatory response and immune cells infiltration in patients with CAVD.

SHC1, a member of SHC family of adaptor proteins, and the role of SHC1 in reactive oxygen species (ROS) production is known to be related to development of atherosclerosis (Tomilov et al., 2010; Miao et al., 2015). Recent evidence suggests that ROS also plays an important role in the pathophysiology of CAVD by inducing the differentiation of valvular stromal cells into myofibroblasts and osteoblasts (Liu et al., 2019).

In this meta-analysis, CIBERSORT algorithm was firstly performed to evaluate the profile of immune cells infiltration in aortic valve tissues from patients with CAVD. We found reduced infiltration of macrophages M2 and NK cells activated, as well as increased infiltration of neutrophils, T cells CD4 memory activated and macrophages M0. Imbalance of M1 and M2 polarization in macrophages is known to be critical in regulating the intensity of inflammatory responses. Our results are identical to previous studies, showing that calcified aortic valves have fewer macrophages M2 compared with aortic valves from normal individuals (Zhou et al., 2020b). In addition, our study has also shown that the macrophages M0 population were significantly elevated in CAVD. Neutrophils and C-reactive protein (CRP) are indirect blood markers that roughly reflect the level of inflammation, which were elevated in calcified aortic valves (Song et al., 2019). Moreover, T cells CD4 memory activated and Tregs was also significantly elevated in patients with CAVD. These results are consistent with previous studies suggesting that calcified aortic stenosis is characterized by chronic inflammation with infiltration of immune cells (Steiner et al., 2012). We also studied the correlation between key DEIRGs and infiltrating immune cells, and found that PTPN1, GRB2, PTPN6, SYK and SHC1 may play a key role in CAVD by modulating immune cells infiltration.

There are several limitations of the present meta-analysis that should be mentioned. Given the age-dependent and gender-dependent clinical features of CAVD, all of the aortic valve samples included in this meta-analysis were derived from elderly male individuals. More aortic valve samples from patients of different regions and ages are needed to investigate the changes in gene expression profile of patients with CAVD. Although 25 aortic valve samples from patients with CAVD and 23 aortic valve samples from normal individuals were included for analysis, it might still be insufficient to identify the key DEGs in CAVD. In addition, the paucity of confirmatory experiments is another significant limitation. It is difficult to obtain aortic valve tissue samples in clinic, especially the aortic valve tissue samples in the control group from normal individuals. We are now trying to overcome the current difficulties in obtaining aortic valve tissue samples. In the near future, we will conduct Next Generation Sequencing (NGS) in the collected aortic valve tissue samples and further study the molecular mechanisms of the occurrence and development of CAVD.

## Conclusion

Above all, we found that *PTPN11*, *GRB2*, *SYK*, *PTPN6* and *SHC1* are key immune related biomarkers of CAVD. Reduced infiltration of macrophages M2 and NK cells activated, as well as increased infiltration of neutrophils, T cells CD4 memory activated and macrophages M0 were found in aortic valve samples from patients with CAVD. Moreover, regulation of *PTPN11*, *GRB2*, *SYK*, *PTPN6* and *SHC1* on immune cells infiltration may play an important role in the occurrence and development of CAVD. Further researches on roles of *PTPN11*, *GRB2*, *SYK*, *PTPN6*, *SHC1* and immune cells infiltration in CAVD are needed whether it might be a new molecular targeted therapy for patients with CAVD.

## Data Availability

The original contributions presented in the study are included in the article/Supplementary Material, further inquiries can be directed to the corresponding author.
