# Supplementary material for: Integrated identification of key immune related genes and patterns of immune infiltration in calcified aortic valvular disease: A network based meta-analysis
Source: Front Genet. 2022 Sep 21;13:971808. doi: 10.3389/fgene.2022.971808 (PMC9532575; doi:10.3389/fgene.2022.971808)
Supplement: Supplementary file 1 [file Table1.DOCX]

**Table 1** Characteristics of the datasets included in the integrated analysis.

| **GEO ID** | **Platform** | **Citation** | **Region** | **Normal** | **CAVD** |
| --- | --- | --- | --- | --- | --- |
| GSE12644 | GPL570; Affymetrix Human Genome U133 Plus 2.0 Array | Bossé Y, et al. Circ Cardiovasc Genet, 2009;2(5):489-498. PMID: 20031625  Derbali H, et al. Am J Pathol, 2010;176(6):2638-2645. PMID: 20382708 | Quebec, Canada | 10 | 10 |
| GSE51472 | GPL570; Affymetrix Human Genome U133 Plus 2.0 Array | Ohukainen P, et al. Ann Med, 2015;47(5):423-429. PMID: 26203686  Rysä J, et al. Genom Data, 2016;7:107-108.  PMID: 26981379 | Oulu, Finland | 5 | 5 |
| GSE83453 | GPL10558; Illumina HumanHT-12 V4.0 expression beadchip | Guauque-Olarte S, et al. Physiol Genomics, 2016;48(10):749-761. PMID: 27495158 | Quebec, Canada | 8 | 10 |

GEO: Gene Expression Omnibus; CAVD: Calcific Aortic Valve Disease.
