# Supplementary figures and images for: Integrated identification of key immune related genes and patterns of immune infiltration in calcified aortic valvular disease: A network based meta-analysis
Source: Front Genet. 2022 Sep 21;13:971808. doi: 10.3389/fgene.2022.971808 (PMC9532575; doi:10.3389/fgene.2022.971808)

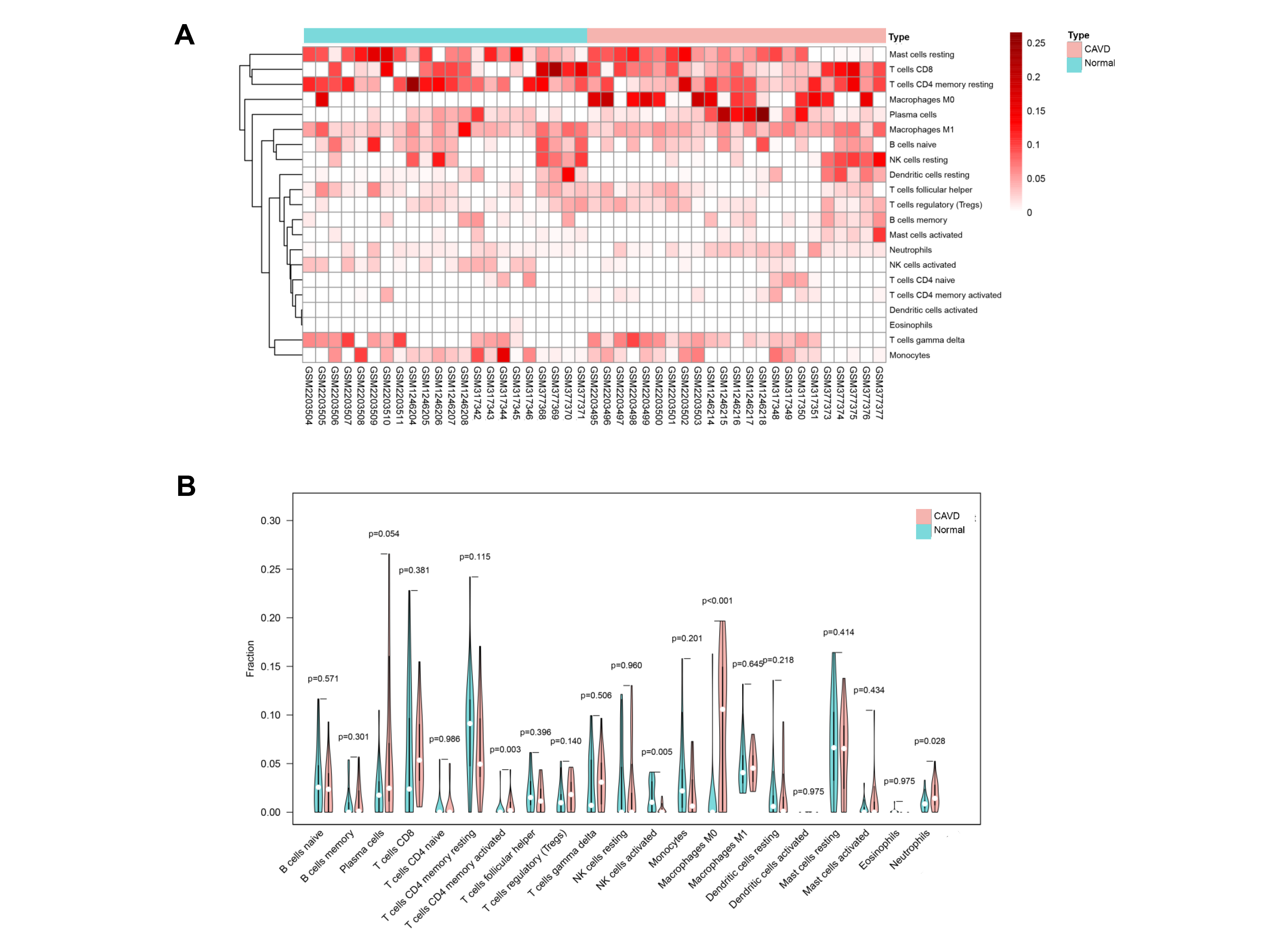

Supplement: Supplementary file 2 [file Image3.TIF]

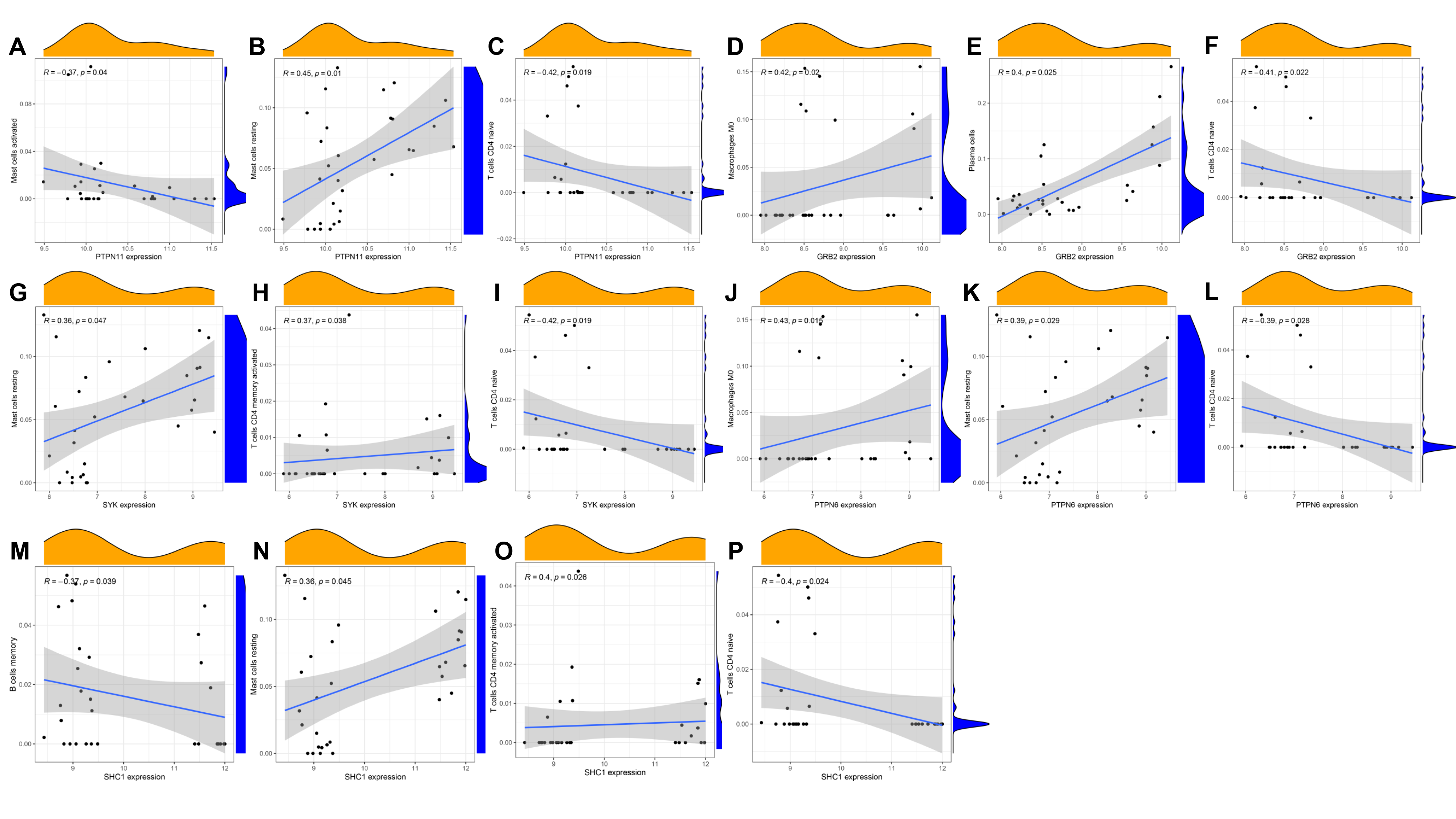

Supplement: Supplementary file 3 [file Image2.TIF]

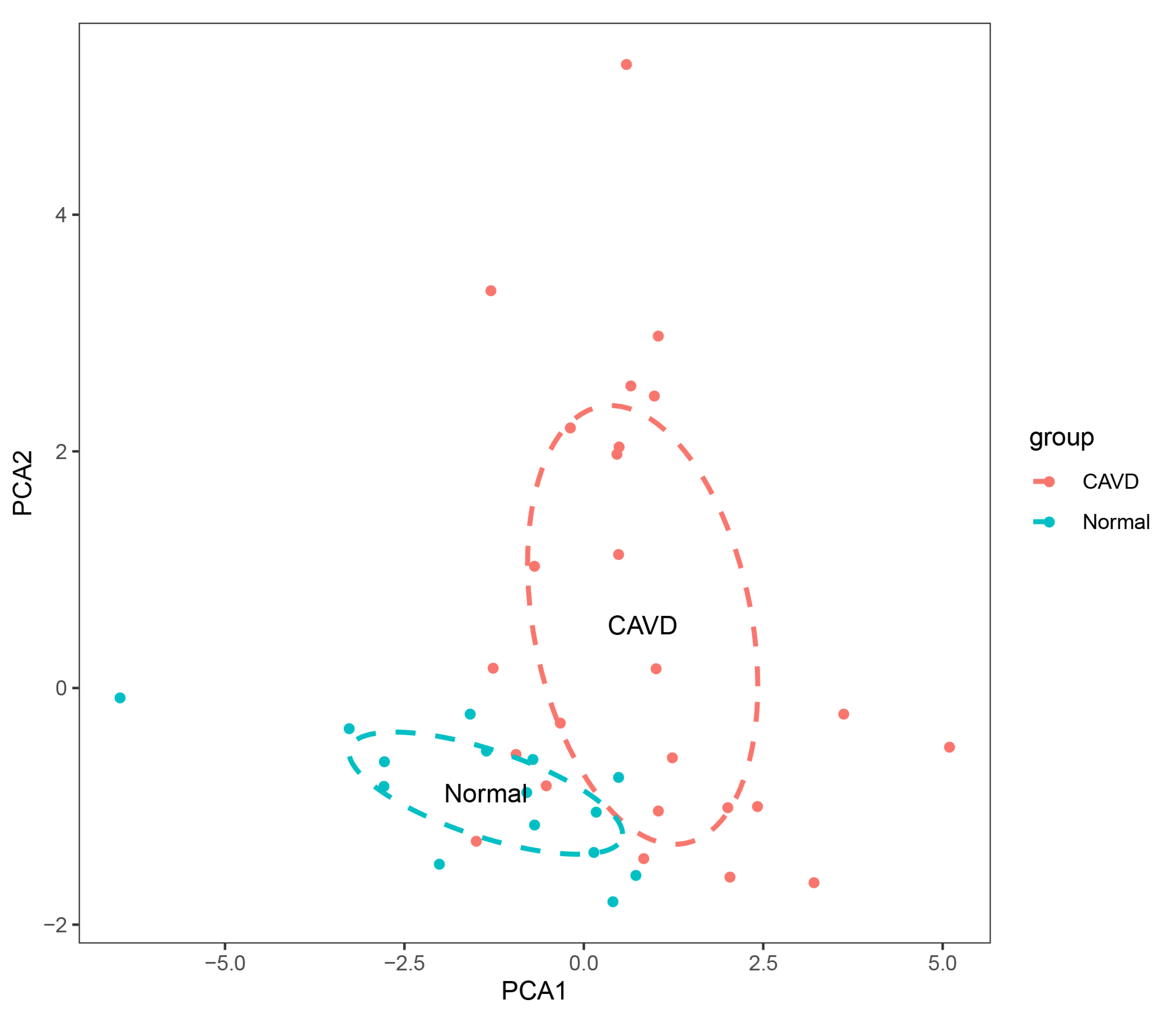

Supplement: Supplementary file 4 [file Image1.TIF]
