# Supplementary material for: Integrated identification of key immune related genes and patterns of immune infiltration in calcified aortic valvular disease: A network based meta-analysis
Source: Front Genet. 2022 Sep 21;13:971808. doi: 10.3389/fgene.2022.971808 (PMC9532575; doi:10.3389/fgene.2022.971808)
Supplement: Supplementary file 5 [file Table2.DOCX]

**Table 2 Summary of the functions and known contributions of key DEIRGs in CAVD and other inflammatory diseases.**

| **Gene symbol** | **Full name** | **Functions and known contributions of key DEIRGs in CAVD and inflammatory diseases** |
| --- | --- | --- |
| *PTPN11* | Protein tyrosine phosphatase 11 | PTPN11 is an important component in growth factor pathway and closely related to formation of valve endothelial cells. Moreover, PTPN11 can reduce the level of Th1 cytokine through preventing combination of STAT1 and IFN-γreceptor. PTPN11 is associated with inflammatory diseases, including pulmonary valve stenosis, ulcerative colitis, inflammation induced-myocardial hypertrophy and cardiac fibrotic remodeling. |
| *GRB2* | Growth factor receptor-bound protein 2 | GRB2 mainly functions in activating Egfr tyrosine kinase and its downstream renin-angiotensin system. GRB2 was also involved in the process of development of T cells and Th cells. Studies have demonstrated that GRB2 was significantly up-regulated in aortic valve tissues form CAVD patients. |
| *SYK* | Spleen-associated tyrosine kinase | SYK is a member of the none receptor type tyrosine kinase family and involved in numerous biological functions. As a proinflammatory molecule, SYK has become a crucial biomarker of coronary heart disease. However, the relationship between SYK and aortic valve diseases still remains exclusive. |
| *PTPN6* | Protein tyrosine phosphatase 6 | PTPN6 specially expressed in the cytoplasm, it can prevent excessive autoimmunity in IL-1 dependent inflammatory diseases. PTPN6 can ameliorate inflammatory diseases by decreasing TNF-α, TGF-β and IL-6 and prevent the harmful effects of pathogens on the host. PTPN6 is known as an important negative regulator of inflammatory response and down regulated in patients with CAVD. |
| *SHC1* | Src-homology 2 domain containing 1 | SHC1 is a member of SHC family of adaptor proteins. SHC1 functions in production of reactive oxygen species. Oxidative stress can cause inflammation and play an important role in the development of CAVD. SHC1 mediated-reactive oxygen species production is closely related to development of atherosclerosis and coronary heart disease. |

CAVD: Calcific Aortic Valve Disease.
